# The prevention of gestational diabetes mellitus (The role of lifestyle): a meta-analysis

**DOI:** 10.1186/s13098-022-00854-5

**Published:** 2022-06-15

**Authors:** Abdullah H. Altemani, Riyadh A. Alzaheb

**Affiliations:** 1 Department of Family and Community Medicine, Faculty of Medicine, University of Tabuk, Tabuk, 71491 Saudi Arabia; 2 Department of Clinical Nutrition, Faculty of Applied Medical Sciences, University of Tabuk, Tabuk, Saudi Arabia

**Keywords:** Diet, Exercise, Lifestyle, Gestational diabetes mellitus, Prevention

## Abstract

Gestational diabetes mellitus (GDM) is the most common complication of pregnancy. The disease is on the rise worldwide with deleterious consequences on the fetus, mother, and children. The study aimed to review the role of lifestyle in the prevention of GDM. We searched PubMed, SCOPUS, Web of Science, Cochrane Library, EBSCO, and Google Scholar from the first published article up to December 2021; articles were eligible if they were controlled trials, prospective cohorts, and case–control. Out of 5559 articles retrieved, 66 full texts were screened, and 19 studies were included in the meta-analysis. (6 studies assessed the effects of diet, and 13 were on exercise). The dietary intervention showed significant positive effect on GDM, odd ratio = 0.69, 95% *CI*, 0.56–84, P-value for overall effect = 0.002. The DASH diet was better than Mediterranean Diet (odd ratio, 0.71, 95% CI, 68–74, P-value < 0.001). Regarding exercise, no significant prevention was evident on GDM, odd ratio, 0.77, 95% *CI*, 0.55–1.06, P-value = 0.11. However, a significant prevention of gestational diabetes was found when the exercise was mild-moderate (odd ratio = 0.65, 95% *CI*, 0.53–80, P < 0.0001) and started in the first trimester (odd ratio, 0.57, 95% *CI*, 0.43–0.75, P < 0.0001. No significant effect was found when the exercise was vigorous (odd ratio = 1.09, 95% *CI*, 0.50–2.38, P = 0.83) and started during the second trimester of pregnancy (odd ratio, 1.08, 95% *CI*, 0.65–1.80, P = 0.77. Diet and early mild-moderate exercise were effective in GDM prevention. Exercise during the second trimester and moderate-vigorous were not. Further studies assessing the type, duration, and frequency of physical activity are needed.

## Background

Gestational diabetes mellitus (GDM) is the most common medical complication of pregnancy, it affects 5–6% of pregnant women in the USA according to the Carpenter-Coustan criteria, and the rate would increase to 15–20% when the International Association of Diabetes in Pregnancy Study Groups criteria is applied. GDM is on the rise due to increasing age and obesity [1]. The rate of obesity and overweight is rapidly increasing globally and in particular, for the Gulf countries including the Kingdom of Saudi Arabia, this is mirrored by the high prevalence of obesity-related disorders including diabetes [2]. Diabetes mellitus is more prevalent among Saudi females mainly due to an unfriendly diet. The rapid development in Saudi Arabia substantially shifted the diet from the healthy traditional diet to a more Westernized diet with deleterious consequences [3]. GDM increases both maternal and fetal complications including excess fetal growth, cardiovascular disease, impaired glucose metabolism, and pregnancy-related hypertensive disease. Physical activity and dietary modifications are the mainstay of management with insulin, Glyburide, and metformin used when normoglycemia is not achieved [4]. Lifestyle modifications need great effort from both the healthcare professionals and the patients and are usually faced with numerous barriers that may lead to poor glycemic control [5]. The role of lifestyle in the prevention of GDM is a matter of controversy. The available evidence regarding the lifestyle modification effects on GDM is weak due to the different diets included; furthermore, no single diet fits all. Mediterranean diet is the only diet that is recommended to patients with diabetes mellitus [6]. Besides, both diet and exercise might be affected by the type, timing, and amount (exercise is also affected by the duration). In addition, non-adherence is a substantial factor compromising the quality of studies [7]. Individual food items were extensively studied, but nutrients are not consumed in isolation either they are consumed in different combinations (dietary patterns). In addition, dietary patterns mimic real-world scenarios and can be translated into simple and easy-to-follow recommendations. Dietary Approaches to Stop Hypertension (DASH), Alternate Healthy Eating Index diet (AHEI), and Mediterranean Diet (MedDiet) were shown to reduce diabetes, mortality, and cardiovascular disease. However, their effects on gestational diabetes prevention are scarce [8–11]. Tobias and colleagues in their retrospective cohort showed that aHEI lowered the risk of GDM by 46%, followed by the DASH diet (34%), and MedDiet (24%) [12]. The previous observation was supported by another study (57%, 46%, and 40% reduction in aHEI diet, DASH diet, and MedDiet respectively) [13]. Another study showed a higher reduction in GDM among patients adherent to MedDiert compared to the DASH diet (80% vs. 71%) [14] A randomized trial supported the above observations and showed the beneficial effects of the DASH diet on glycemic and lipid parameters [15]. Further randomized controlled trials showed the beneficial effects of the DASH diet on insulin resistance and glycemic parameters [16]. To the best of our knowledge, no review compared different dietary patterns in the prevention of gestational diabetes mellitus. Therefore, the current review assessed the effects of MedDiet, DASH diet, and aAEI diet on the prevention of gestational diabetes and assessed if one diet is superior. In addition, this meta-analysis assessed the effectiveness of exercise (throughout, first and second trimester).

## Methods

### Articles selection according to PICOS

We searched PubMed, SCOPUS, Web of Science, Cochrane Library, EBSCO, and the first 100 articles in Google Scholar from the first published article up to December 2021, articles were eligible if they were controlled trials, prospective cohorts, and case–control studies and published in English. Article in languages other than English, other methodologies (case series, and case reports were not included. The trials must fulfill the following outcomes to be included:

- The effect of the Mediterranean diet, Dietary Approaches to Stop Hypertension (DASH), Alternate Healthy Eating Index diet (AHEI) on GDM prevention
- The effect of first and second-trimester exercise on GDM prevention.

We excluded diabetes mellitus prevention programs carried on patients with type 2 diabetes mellitus or women with established GDM.

### Literature search and data extraction

**Fig. 1:**
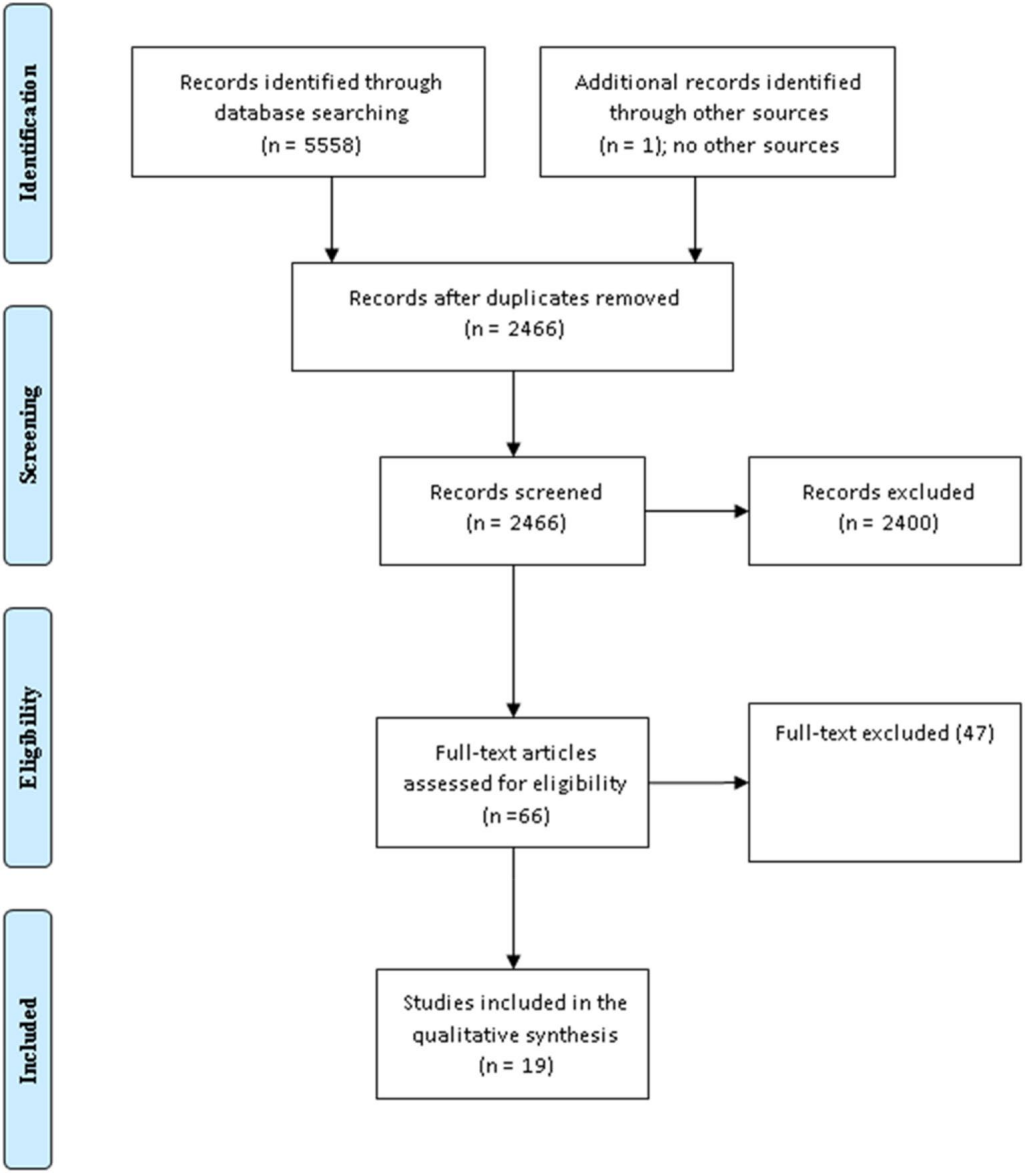
Exercise and diet in the prevention of gestational diabetes (The PRISMA Chart)

The two authors (A.H, and R.A) searched the mentioned databases for relevant articles, out of 5559 articles retrieved, 66 full texts were screened, and 19 studies were included in the meta-analysis. Fig. 1. In the current review, 6 studies assessed the effects of diet, and 13 were on exercise. Of them, eight assessed exercises in the first trimester and five in the second trimester. We did not specify any criteria for GDM diagnosis due to the different methods that might be applied during the long period of the search engine. The retrieved data were exported to an excel sheet detailing the author's names, country of origin of the study, the number of patients and control subjects, and the total number of events in the interventional and exercise groups (Tables 1 , 2). The details of exercise including the type, duration, and intensity, the diet type, and the compliance when reported were also recorded. In this review we concentrated on dietary habits and detailed exercise (timing and duration). Tables 3 ,4 and Fig. 1.

**Table 1:** A comparison between Mediterranean diet, DASH diet, and aAEI diet in reducing gestational diabetes incidence

Author	Year	Country	Duration	Methods	MedDiet	DASH diet	aAEI	Results
Tobias et al	2012	USA	10 years	Prospective	3660/15,254	5186/15,254	7016/15,254	aAEI superior
Tobias et al	2012	USA	14 years	Prospective	2109/52 743	2426/52 743	3006/52 743	aAEI superior
Izadi et al	2016	India	Case–control	Case–control	160/200	142/200	Not assessed	MedDiet better

**Table 2:** GDM outcomes dichotomous variables

Author	Outcomes interventional	Outcomes control	Intervention type	Results
Assaf-Balut et al. 2017 [17]	74/434	103/440	Med Diet	Significant reduction, P = 0.05
HA I Wattar B et al. 2019 [18]	84/477	124/497	MedDiet	Significant reduction P = 01
Sahariah SA et al. 2016 [19]	44/492	57/516	Local diet	Non-significant, P = 0.27
Barakat R et al. 2019 [20]	6/234	15/222	Exercise	Significant reduction, P = 0.033
Cordero Y et al. 2015 [21]	1/101	13/156	exercise	Significant reduction, P = 0.009
da Silva G et al. 2017 [22]	16/205	31/470	Exercise	Non-significant
Daly et al. 2017 [23]	25/44	21/44	Exercise	Non-significant, P = 0.51
Gallaway et al. 2010 [24]	5/22	3/19	Exercise	Non-significant, P = 0.29
Nobles C et al. 2015 [25]	12/124	19/127	Exercise	Non-significant, P = 0.20
Oostdam et al. 2012 [26]	7/62	11/59	Exercise	Non-significant
Ruiz et al. 2013 [27]	7/335	18/352	Exercise	Non-significant
Seneviratne et al. 2016 [28]	4/38	2/37	Exercise	Non-significant
Simmons et al. 2016 [29]	30/110	35/105	Exercise	Non-significant
Stafine et al. 2012 [30]	25/325	18/327	Exercise	Non-significant, P = 0.52
Tomic et al. 2013 [31]	3/168	14/168	Exercise	Non-significant
Wang C et al. 2017 [32]	33/150	61/150	Exercise	Significant reduction, P = < 0.001

**Table 3:** Moderate-strong physical activity effects on gestational diabetes prevention

Author	Exercise type	Duration	Intensity	Results
Stafine et al	Aerobic, three or more times,	From week 18, poor compliance, 55%	Moderate-high	Not sig
Cordero et al. [21]	Aerobic, three times	50–60 min throughout pregnancy	strong	Improved glucose intolerance
Daly et al. [23]	Aerobic and resistance	50–60 min throughout pregnancy	strong	Not sig
Callaway et al. [24]	Energy expenditure goal of 900 kcal/week	Energy expenditure goal of 900 kcal/week	Moderate-vigorous	Not sig

**Table 4:** Mild-moderate physical activity effects on gestational diabetes prevention

Author	Exercise type	Duration	Intensity	Results
da Silva et al. [22]	Aerobic and resistance, three times	60 min, from week 16, overweight	Moderate	Not sign
Nobles et al. [25]	Aerobic, most days	30 min, from 12 weeks, overweight	Moderate	Not sig
Oostdam et al. [26]	Aerobic and resistance, three times	Duration not stated, from 12 weeks, overweight	Moderate	Not sig
Seneviratne et al. [28]	Aerobic, cycling	Compliance was poor, 33%, from 20 weeks, overweight	Moderate	Not sig
Barakat et al. [20]	Aerobic, three times	50–55 min throughout pregnancy	Moderate	Improved glucose intolerance
Ruiz et al. [27]	Aerobic and resistance, three times	50–55 min throughout pregnancy	Mild-moderate	Improved glucose intolerance
Simmons et al. [29]	Aerobic and resistance daily	Throughout pregnancy, no specific duration	Mild-moderate	Not sig
Tomić et al. [31]	Aerobic, three times	50 min throughout pregnancy	Moderate	Improved glucose intolerance
Wang et al. [32]	Aerobic, three times	30 min of cycling throughout pregnancy	Moderate	Improved glucose intolerance

### Quality assessment of the cited trials

The quality of the included studies was assessed using a modified Cochrane risk of bias. Table 5.

**Table 5:** Risk of bias of the included randomized controlled trials

Study	Year	Selection	Performance	Attrition	Reporting	Other
Assaf-Balut et al. [17]	2017	Low	High	Low	Low	Low
H Al Wattar et al. [18]	2019	Low	High	Low	Low	Low
Sahariah et al. [19]	2016	Unclear	High	Low	Low	Low
Barakat et al. [20]	2019	Low	Low	low	High	Unclear
Cordero al. [21]	2015	High	Unclear	High	Low	Unclear
da Silve et al. [22]	2017	low	Unclear	Low	Low	Low
Daly et al. [23]	2017	Low	Unclear	High	Low	Low
Callaway et al. [24]	2010	Low	Unclear	High	Low	Low
Nobles et al. [25]	2015	High	Low	Unclear	Low	Low
Oostdam et al. [26]	2012	High	High	Low	High	Low
Ruiz et al. [27]	2013	Low	Unclear	Nuclear	High	Unclear
Seneviratne et al. [28]	2016	Unclear	Low	Low	Low	Low
Simmons et al. [29]	2017	Low	High	Low	Low	Low
Stafne et al. [30]	2012	Low	Low	Low	High	Unclear
Tomić et al. [31]	2013	High	Unclear	High	Unclear	Unclear
Wang et al. [32]	2017	Unclear	High	Low	High	Low

### Data analysis

We use RevMan (version 5, 4) for data analysis, the data were entered manually, and dichotomous variables were compared. The fixed effect was applied unless a significant heterogeneity (> 50%) was observed. A P-value of 0.05 is significant except when a significant heterogeneity.

### Ethical consideration

We did not include studies published by the authors.

## Results

**Fig. 2:**
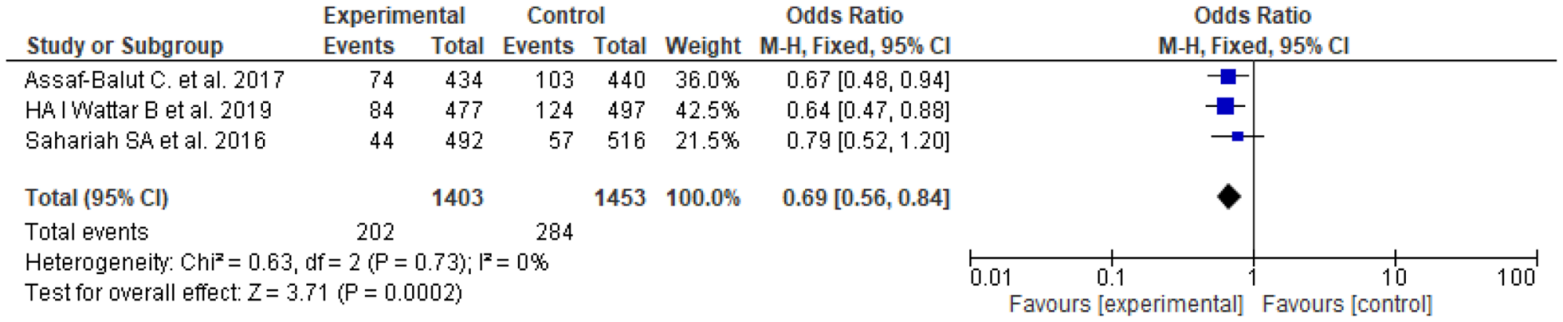
The effects of diet on gestational diabetes prevention

The dietary intervention included three studies [18, 19] with significant positive effect on GDM, odd ratio = 0.69, 95% *CI*, 0.56–84, P-value for overall effect = 0.002, with no significant heterogeneity, *I*^*2*^ = 0.0%. Fig.  2.

**Fig. 3:**
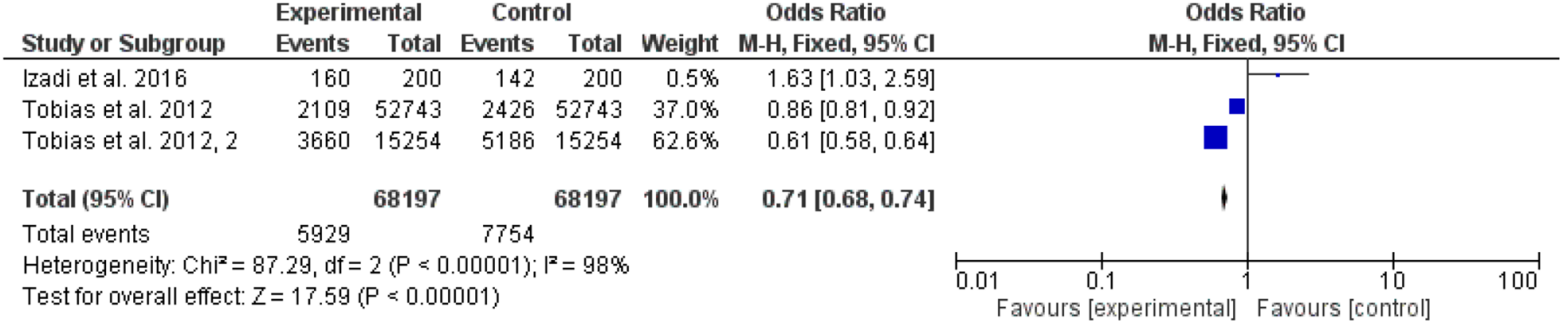
A comparison between Dietary Approach to Stop Hypertension (DASH) and Alternate Healthy Eating Index diet (AHEI)effects on gestational diabetes

**Fig. 4:**
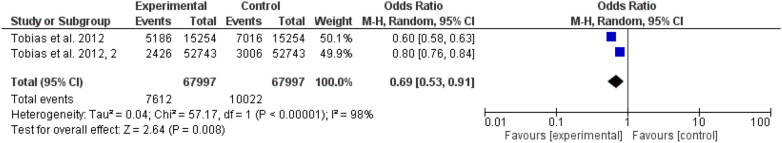
A comparison between Dietary Approach to Stop Hypertension (DASH) and Mediterranean diet effects on gestational diabetes

The DASH diet showed superiority to MedDiet Mediterranean Diet [13–15] (odd ratio, 0.71, 95% CI, 68–74, P-value < 0.001). Furthermore, the Alternate Healthy Eating Index diet was better than the DASH diet (odd ratio, 0.69, 95% CI, 53–91, P-value, 0.008). Fig. 3 and 4.

**Fig. 5:**
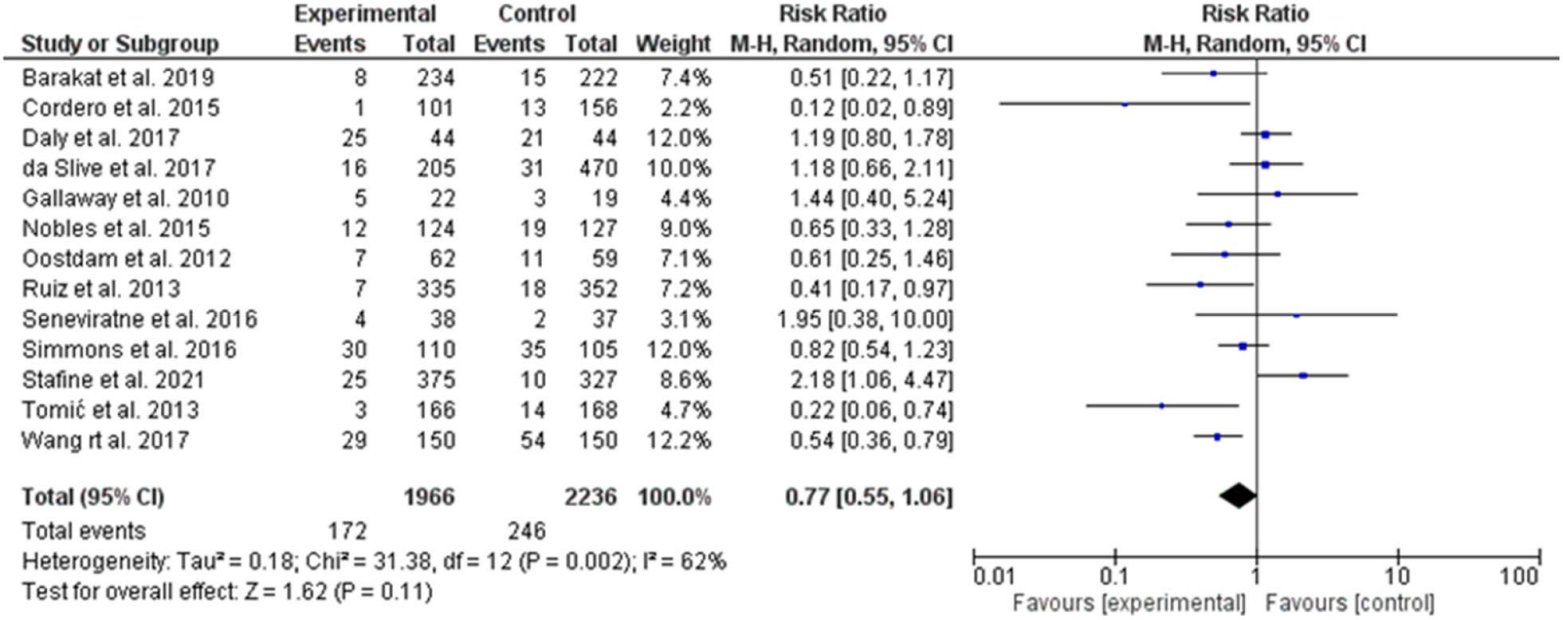
The effects of exercise on gestational diabetes prevention

Regarding exercise, there were thirteen studies [20–32] with no significant prevention on GDM (total patients 4202 and 418 events), odd ratio, 0.77, 95% *CI*, 0.55–1.06, P-value for overall effect = 0.11, with significant heterogeneity, *I*.^*2*^ = 62% and P-value for heterogeneity, 0.002. Fig.  5

**Fig. 6:**
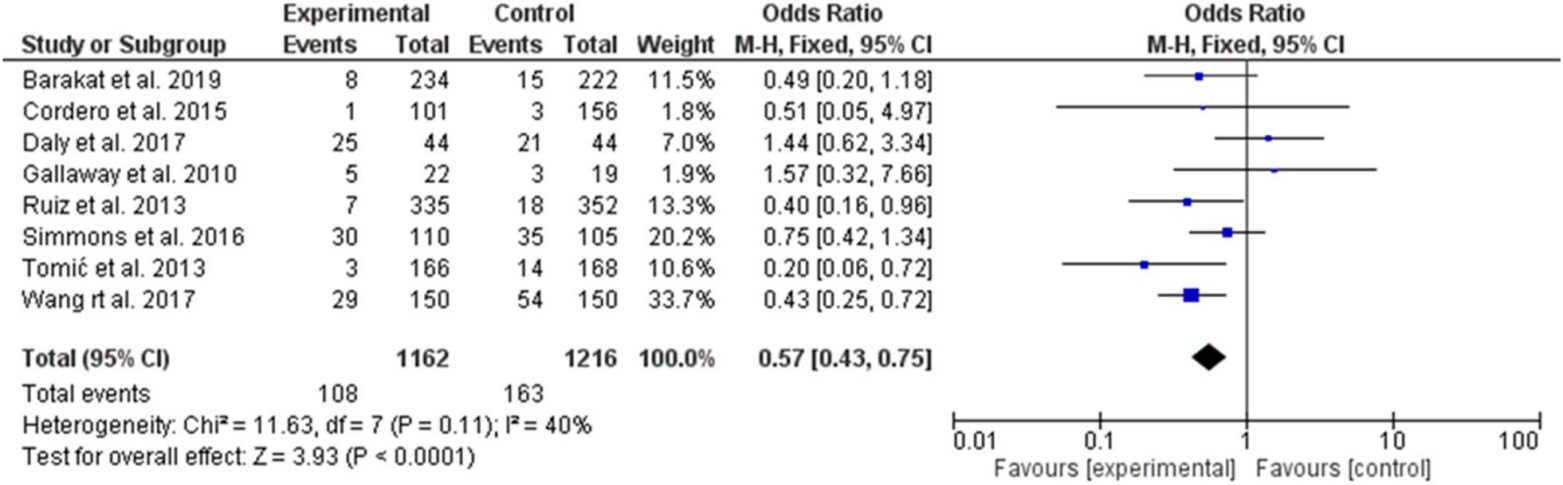
The effects of exercise during the first trimester of pregnancy on gestational diabetes prevention

However, when a sub-analysis was conducted, significant prevention of gestational diabetes was found when the exercise started in the first trimester (odd ratio, 0.57, 95% *CI*, 0.43–0.75, P-value for overall effect < 0.0001, with significant heterogeneity, *I*^*2*^ = 40%. Fig.  6.

**Fig. 7:**
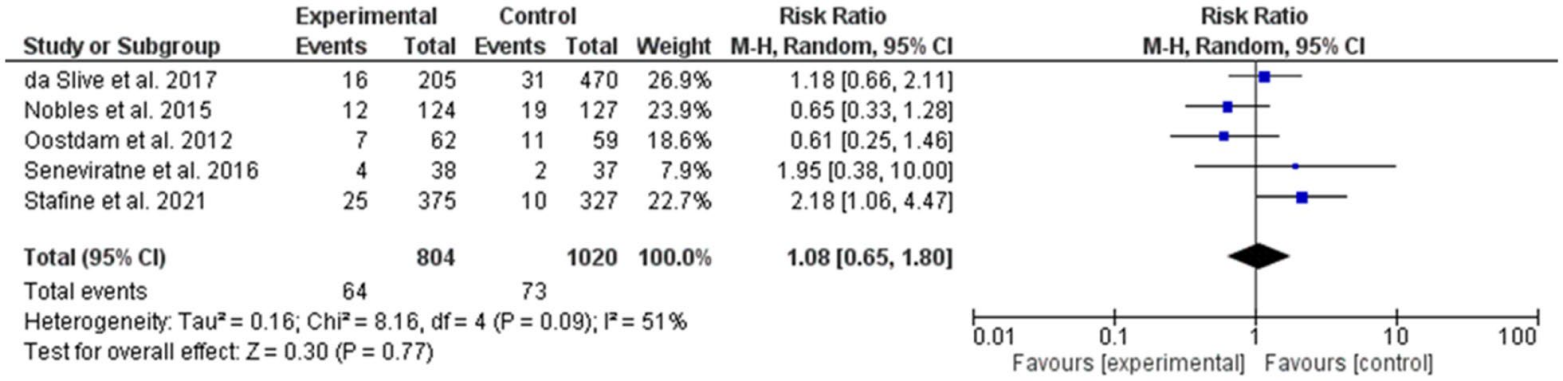
The effects of exercise during the second trimester on gestational diabetes prevention

No significant effect was found when the exercise started during the second trimester of pregnancy [22, 25, 26, 28, 30] (odd ratio, 1.08, 95% *CI*, 0.65–1.80, P-value for overall effect = 0.77, with significant heterogeneity, *I*^*2*^ = 51%. Fig. 7.

**Fig. 8:**
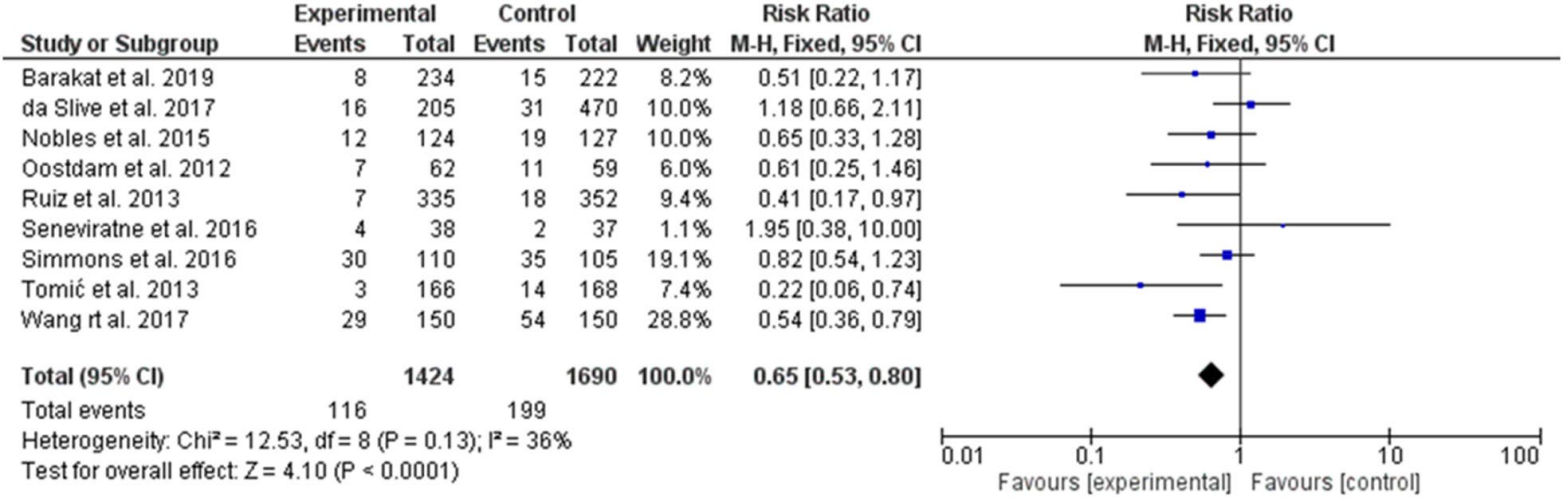
The effects of mild-moderate exercise on gestational diabetes prevention

**Fig. 9:**
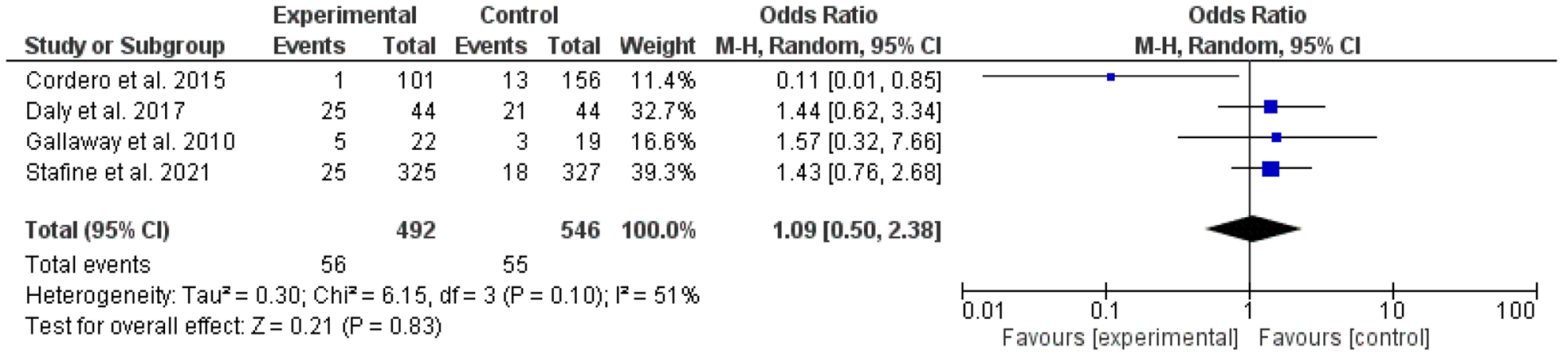
The effects of vigorous exercise on gestational diabetes prevention

It is interesting to note that mild-moderate intensity exercise was effective in gestational diabetes prevention (odd ratio = 0.65, 95% *CI*, 0.53–80, P-value for overall effect < 0.0001, with significant heterogeneity, *I*^*2*^ = 36.0%. Fig. 8), while vigorous activity was not (odd ratio = 1.09, 95% *CI*, 0.50–2.38, P-value for overall effect = 0.83, with no significant heterogeneity, *I*^*2*^ = 51.0%. Fig. 9).

## Discussion

The present meta-analysis showed that diet was effective in gestational diabetes prevention, while exercise was not. However, a sub-analysis showed that exercise was effective only when introduced in the first trimester; physical activity started in the second trimester was not effective. In addition, mild-moderate exercise was effective in contrast to vigorous physical activity. Previous studies with a low quality of evidence showed the efficacy of combined diet and exercise [33, 34]. The meta-analyses lack a face-to-face comparison between different forms of diets. In addition, they did not report the timing and intensity of exercise. Another study showed similar results but only among obese women [35]. Dietary supplementation with myo-inositol reduced GDM in a study that included four trials and lacked generalization [36].

## Special types of diets

### Alternate healthy eating index-2010

The AHEI-2010 is a measure of diet quality based on food items predictive of major chronic disease risk, particularly cardiometabolic disease including stroke and diabetes, and malignancies. It emphasizes a high intake of legumes and nuts, cereals, vegetables and fruits, omega-3 fats, and polyunsaturated fatty acids while limiting the intake of red and processed meats, sodium, sugary beverages, and alcohol [37].

The effect of the Alternate Healthy Eating Index-2010 in reducing GDM ranged from 19–46%, interestingly, when the diet is combined with other risk factors reduction (physical activity, smoking, and normal body weight) the risk reduction may be as high as 83% [38]. A 41% lower risk of gestational diabetes was observed among patients adherent to aHEI [39]. A genetic interaction increases the like hood of developing GDM among patients on aHEI diet [40]. In addition, health education and self-efficacy substantially improved the quality of aHEI [41], the included studies showed that Alternate Healthy Eating Index is superior compared to the DASH diet and MedDiet [13–15]**.**

### Mediterranean diet (MedDiet)

The Mediterranean diet is a diet with high fruits and vegetables, bread, legumes, olive oil, cereals, fish, and limited animal products [42].

The Mediterranean diet is promising for the prevention of GDM, however, the results are contradictory. A recent meta-analysis of the randomized controlled trial showed the efficacy of MedDiet in preventing GDM [43]. A recent review examined the role of MedDiet in modulating immune response and inflammation during COVID-19 and showed that MedDiet reduced interleukin-6 and inflammatory markers [44]. A recent case–control study showed that MedDiet reduced GDM incidence [45], interestingly, women who have rs7903146 T-allele showed a high reduction of GDM compared to their counterparts who are not indicating a gene-diet interaction [46]. The protective effect of MedDiet ranged from 15 to 38% depending on compliance, genetic factors, and the diagnostic method used (8% when the American Diabetes Association was used and 24% If The International Association of the Diabetes and Pregnancy Study Groups was used [38]. Although MedDiet was effective in preventing gestational diabetes mellitus, DASH diet and aAEI showed superiority [13–15].

### Dietary approaches to stop hypertension (DASH)

Dietary Approaches to Stop Hypertension (DASH) originally developed for hypertension consists of high intakes of fruits, vegetables, legumes, and nuts, moderate low-fat dairy products, and low consumption of sodium, animal protein, and sweets [47], of the three studies investigating the effects of the DASH diet on gestational diabetes showed superiority when compared to MedDiet. However, the DASH diet was inferior to Alternate Healthy Eating Index [13–15].

### Western and prudent diet

Zhang and colleagues found a positive association between Western and a negative association of prudent diet with GDM risk, while Radesky et al. found no associations. [48, 49].

### Physical activity

In the present study, exercise was effective in the first trimester and not during the second trimester; previous studies showed lifestyle before gestation was effective in reducing diabetes. However, exercise during pregnancy was not [50]. Other studies showed early pregnancy (before the 15^th^ week) lifestyles were effective [51] in line with our findings. The effect of physical activity on GDM prevention depends on the time and duration of physical activity. Exercise before or in early pregnancy was effective in reducing GDM in the majority of studies [52–55], while few studies showed no significant reduction. [56, 57]. A leisure time of physical activity of 150 h/week before pregnancy was associated with 68% risk reduction, and the benefits increased with longer duration [57].

Davenport and colleagues found that exercise of moderate intensity is effective in the prevention of GDM when adopted prenatally in line with our findings [58], the same conclusion was shown by other studies [59]. Davenport et al. study was limited by including all types of articles except case studies. A meta-analysis from China showed that diet and exercise were effective in reducing GDM when introduced earlier (before the 15th week), an effect that was not robot after that [51]. Other studies published in Spain and Australia [9, 60] and concluded the effectiveness of dietary advice and moderate exercise combination in GDM prevention in contradiction to the present result, a plausible explanation might be the different methods of exercise and the type of diet adopted. The earlier adoption of 50–60 min of moderate physical activity and targeting at-risk women with a high body mass index was found to be more effective [61]. However, an update of the same study showed a limited ability to inform practice due to the risk of bias observed in the included studies [62]. Further studies limited by including retrospective, cross-sectional, and case–control studies support the above findings regarding exercise [63]. We found only one meta-analysis assessing the effect of early and late exercise on GDM. However, the small number of included studies and the high heterogeneity limited the studies [64]. The study found no effect throughout pregnancy. A non-linear negative association between exercise before and during early pregnancy was observed by the previous literature. The association was steeper at lower levels; however, the benefits plateaued at 8–10 h a week, a finding that may explain the contradiction between various studies assessing the effects of exercise on GDM [24]. The compliance to exercise, duration, timing, and type of exercise might greatly influence the outcomes. To best inform Obstetric guidelines, studies putting the previous parameters into consideration are warranted.

The strength of the current meta-analysis is that it is the first to compare different types of diets and define the time, intensity, duration, and frequency of exercise.

### Limitations

The study limitations were including some observational studies, the limitations to the English language, and the significant heterogeneity observed.

## Conclusion

The Dietary Approaches to Stop Hypertension, Alternate Healthy Eating Index diet, and Mediterranean Diet were effective in reducing gestational diabetes mellitus. The DASH diet showed superiority to the Mediterranean. Furthermore, the Alternate Healthy Eating Index diet was better than the DASH diet. Data regarding physical activity were conflicting. Early mild-moderate physical activity was effective, while late, moderate-vigorous exercise was not. Randomized control trials and genetic studies are needed for the individualization of dietary patterns.

## Data Availability

The data used in this manuscript are available upon request.

## Abbreviations

MedDiet: Mediterranean diet
DASH diet: Dietary Approaches to Stop Hypertension
AHEI: Alternate Healthy Eating Index diet
GDM: Gestational diabetes mellitus
